# A 5-Methylcytosine Site of Growth Differentiation Factor 9 (GDF9) Gene Affects Its Tissue-Specific Expression in Sheep

**DOI:** 10.3390/ani8110200

**Published:** 2018-11-07

**Authors:** Zhangyuan Pan, Xiangyu Wang, Ran Di, Qiuyue Liu, Wenping Hu, Xiaohan Cao, Xiaofei Guo, Xiaoyun He, Shengjin Lv, Fukuan Li, Hui Wang, Mingxing Chu

**Affiliations:** 1 Key Laboratory of Animal Genetics and Breeding and Reproduction of Ministry of Agriculture, Institute of Animal Science, Chinese Academy of Agricultural Sciences, Beijing 100193, China; pzq170450077@163.com (Z.P.); xiangyu_wiggle@163.com (X.W.); dirangirl@163.com (R.D.); qiuyue1983921@163.com (Q.L.); pinkyhoho@163.com (W.H.); caoxiaohan2007@hotmail.com (X.C.); guoxfnongda@163.com (X.G.); hexy2015x@163.com (X.H.); 2 College of Agriculture and Forestry Science, Linyi University, Linyi 276000, China; lvshenjin@lyu.edu.cn (S.L.); lifukuan@lyu.edu.cn (F.L.); wanghui0512@lyu.edu.cn (H.W.)

**Keywords:** *GDF9*, methylation, mRNA expression, tissue-specific, regulatory mechanism

## Abstract

**Simple Summary:**

Growth differentiation factor 9 (GDF9) is an important gene for ovine fertility. GDF9 is highly expressed in the ovary as opposed to other tissues, but the reason for this is unknown. Our study found this can be caused by the methylation level of the promoter CpG island mC-4 site. This finding contributes to the understanding of the regulatory mechanism of GDF9 gene in reproduction.

**Abstract:**

Growth differentiation factor 9 (*GDF9*) plays an important role in the early folliculogenesis of sheep. This study investigated the mRNA expression of ovine *GDF9* in different tissues by real-time PCR. *GDF9* exhibits significantly higher levels of expression (*p* < 0.01) in the ovary, relative to other tissues, indicating that its expression is tissue specific. To explore the regulatory mechanism of this tissue-specific expression, the methylation level of one CpG island (−1453 to −1854) of *GDF9* promoter in ovary and heart was determined. In this region (−1987 to −1750), only the mC-4 site was present in the Sp4 binding site showed differential methylation between the heart and ovary; with increased (*p* < 0.01) methylation being observed in the heart. Additionally, the methylation level was negatively correlated with *GDF9* mRNA expression (R = −0.75, *p =* 0.012), indicating that the methylation of this site plays an important role in transcriptional regulation of *GDF9*. The methylation effect of the mC-4 site was confirmed by using dual-luciferase. Site-directed mutation (methylation) of mC-4 site significantly reduced (*p* < 0.05) basal transcriptional activity of *GDF9* promoter in oocytes. These results imply that methylation of *GDF9* promoter CpG island mC-4 site may affect the binding of the Sp4 transcription factor to the *GDF9* promoter region in sheep, thereby regulating *GDF9* expression and resulting in a tissue-specific expression.

## 1. Introduction

Growth differentiation factor 9 (GDF9) is a member of the transforming growth factor beta superfamily and was first identified in the human ovary [1]. In *GDF9*-deficient female mice, follicular development is halted at the single-layer primary follicle stage, causing infertility [2]. In sheep, *GDF9* mutations (*FecG^H^*, *FecG^T^*, *FecG^E^*, *FecG^F^*, and *FecG^V^*) resulted in hyperprolificacy in heterozygotes and sterility in homozygotes [3,4,5,6,7,8]. Furthermore, a recent study discovered highly heritable markers within *GDF9*, which are important in determining prolificacy traits in sheep [9]. Thus, *GDF9* is an important candidate gene in ovine fertility.

*GDF9* is highly expressed in the ovaries of many species [10,11,12,13,14]. In sheep, *GDF9* expression was higher in both fetal and adult ovaries when compared to expression in other tissues, indicating that *GDF9* is tissue-specific [15,16,17]. *GDF9* also followed a stage-specific pattern of expression during the in vivo development of ovarian follicles in sheep [18,19]. Although several studies have investigated the role of ovine *GDF9* in ovarian function [20,21] and the transcriptional regulation of a mutation of *GDF9* in the ovaries of Fetal sheep [22], the underlying transcriptional mechanisms leading to the tissue-or stage-specific expression of *GDF9* in sheep remain unclear.

Gene promoters are critical cis-regulatory elements for gene transcription that can drive tissue-specific gene expression [23,24]. The methylation of a promoter sequence is an important type of epigenetic control of gene expression [25]. Moreover, the methylation of a gene promoter may result in tissue-specific gene expression [26,27]. In a recent study, we cloned and analyzed the ovine *GDF9* 5′ flanking sequence and found that it included a CpG island [28]. Then, to determine whether the methylation of this CpG island can lead to tissue-specific expression. In this study, *GDF9* mRNA levels in ten tissues were verified using real-time PCR, after which we performed a dual-luciferase assay and site-directed mutation analysis to investigate the relationship between the tissue-specific expression of *GDF9* and the methylation of a CpG island in the *GDF9* promoter. Our results provide important insight into the regulatory mechanism of *GDF9*.

## 2. Materials and Methods

### 2.1. Animals

Small Tail Han (STH) sheep is a famous prolific breed, and the litter size can exceed 2.61 [29]. Five Small Tail Han (STH) ewes were raised on the same farm in Ningxia Hui Autonomous Region, China. Healthy sheep (3–4 years of age) were slaughtered after evidence of estrous in response to teaser rams. Tissues were harvested, snap-frozen in liquid nitrogen, and then stored at −80 °C. Ten tissues samples (heart, liver, lung, spleen, uterus, oviduct, ovary, cerebellum, pituitary, and hypothalamus) were collected from each animal for tissue expression and DNA methylation analysis.

The experimental procedures were approved by the Institute of Animal Sciences, Chinese Academy of Agricultural Sciences (IASCAAS-AE-03).

### 2.2. Detection of GDF9 Expression by Real-Time PCR 

RNA extraction was performed following the manufacturer’s instructions using TRIzol reagent (TaKaRa, Dalian, China), and was treated with DNase using a TURBO DNA-free Kit (Ambion, Austin, TX, USA). The cDNA was generated by PrimeScriptTM^RT^ reagent kit (TaKaRa, Dalian, China).

*GDF9* primers (Exon-span) for real-time PCR were designed according to NM_001142888.2 using Primer-BLAST (NCBI). 20 μL of reaction mixture of real-time PCR including cDNA (2 µL), 10 μM each primer (0.4 µL), ROX Reference Dye II (50×) (0.4 µL), SYBR Green Master Mix (2×) (10 µL), and ddH_2_O (6.8 µL). Reactions were performed under the following conditions: 30 s of 95 °C, followed by 40 cycles of 95 °C for 5 s and 34 s of 60 °C. Negative control reactions were carried out without template. Each reaction was performed in triplicate wells. The amplification efficiency was evaluated using a standard curve. Table 1 lists the primers that were used for real-time PCR. *GAPDH* served as the normalization control.

### 2.3. Sequence Analysis of GDF9 5′-Flanking Sequence

The 2304 bp 5′-flanking sequence of *GDF9* has been validated in a recent study [28]. The CpG islands were predicted using MethPrimer (http://www.urogene.org/cgi-bin/methprimer/methprimer.cgi). The transcription binding site was analyzed with Matinspector (solution parameters: core similarity 1.0; matrix-optimised) (Table 2) and Signal Scan (https://www-bimas.cit.nih.gov/molbio/signal/). 

### 2.4. DNA Isolation and Bisulfite Treatment

Heart and ovary tissues from each of the five STH sheep were used for methylation analysis. Genomic DNA was isolated by QIAamp DNA Mini Kit (QIAGEN, Valencia, CA, USA), and 1 μg of DNA was converted using the EpiTect Bisulfite Kit (QIAGEN, Valencia, CA, USA), according to the manufacturer’s manual. After the chemical conversion, each unmethylated cytosine was converted to an uracil, whereas each methylated cytosine was protected.

### 2.5. Bisulfite Sequencing

The methylation status of one CpG island (−1854, −1453) of *GDF9* was analyzed by bisulfite sequencing PCR (BSP) method. The BSP primers were designed by the MethPrimer program (http://www.urogene.org/methprimer/) (Table 1). The reaction volume of 20 μL contained 1 μL of bisulfite-treated genomic DNA, 10 μL of *Taq* master mix, 8 μL of ddH_2_O, and 0.5 μL each of forward and reverse primers (10 μM). The PCR conditions were as follows: 95 °C for 8 min, 34 cycles of 95 °C for 30 s, 55 °C for 30 s, 72 °C for 30 s, and 72 °C for 8 min. Next, the PCR products were cloned into the pMD18-T vector (TaKaRa, Dalian, China), and 10 positive clones from each tissues of each animals were sequenced (Invitrogen, Shanghai, China).

### 2.6. GDF9 Promoter Deletion Constructs 

The regulatory region of 2636 bp fragment of *GDF9* has been sequenced in a previous study (Pan et al. 2016). Primers were designed to amplify six fragments of this region. The P1 promoter fragment was obtained using P1F and PR primers (Table 1). A *Kpn*I restriction site was added to the 5′ end of the forward primer, whereas a *Hind*III restriction site was added to the 5′ end of the reverse primer. The PCR products were cloned into the pMD18-T vector (TaKaRa). Thereafter, they were excised with *Kpn*I and *Hind*III (NEB) and subcloned into the promoterless pGL3-basic vector (Promega, Madison, WI, USA). The recombinant constructs were designated as pGL3-basic-P1 (−1/−228), pGL3-basic-P2 (−1/−591), pGL3-basic-P3 (−1/−899), pGL3-basic-P4 (−1/−1299), pGL3-basic-P5 (−1/−1750), pGL3-basic-P6 (−1/−1987), and pGL3-basic-P7 (−1/−2277). Subsequently, the constructs were transfected into oocytes to detect luciferase activity.

### 2.7. Site-Directed Mutation of Sp4 Binding Element

A putative Sp4 transcription factor-binding site (−1790 bp) within the pGL3-Basic-P6 was mutated by Quick Change Lightning SDM kit (Stratagene, Santa Clara, CA, USA). Primer 5′ TCTGGGGTCCCGGGGAGCCCCCCACCGGATCC 3’ with complementary reverse primer were used for PCR amplification of pGL3-Basic-P6-mut1. The resultant mutation was confirmed by sequencing. Then the constructs were transfected into oocytes to detect luciferase activity.

### 2.8. In Vitro-Methylation

According to the manufacturer’s instructions of methylase *M.Sss*I (New England Biolabs), two plasmids with methylated CpG promoters, pGL3-Basic-P6-methylation and pGL3-Basic-P5-methylation were generated. The methylation status was verified by methylation-sensitive restriction eyzyme *NarI* (New England Biolabs). After purification with QIAquick Nucleotide Removal Kit (QIAGEN, Valencia, CA, USA), the plasmids were transfected into oocytes to detect luciferase activity.

### 2.9. Luciferase Assay

According to the method in a previous report [30], a dual luciferase assay was performed in ovine oocytes. Ovaries were collected from a local abattoir, stored in PBS buffer (including streptomycin sulphate 100 μg/mL and penicillin 100 U/mL) on ice, and transported to the laboratory within 4 h. Next, the ovaries were washed three times in normal saline, and follicular fluid of large antral follicles (3–6 mm) was collected from the visible follicles with a sterile injector. Only cumulus oocyte complexes (COCs) with more than five layers of cumulus mass were selected. Pools of 30 COCs were incubated in standard medium (DMEM with 15% FBS, 0.005% streptomycin, 0.005% penicillin) for 12 h. Finally, the COCs were treated with hyaluronidase and pronase, and oocytes were collected into fresh standard medium and used for transient transfection. 

After 12 h of cultured, promoter luciferase reporter constructs were transfected into oocytes by Lipofectamine™ 3000 (Invitrogen, Carlsbad, CA, USA). A GFP reporter vector with CMV promoter were used to verify that the efficiency of transfection was greater than 85%. For dual luciferase assay, each well was transfected with a 3:1 ratio of Lipofectamine™ 3000 transfection reagent to total plasmids which included a 20:1 ratio of luciferase plasmids to PRL-TK vector (Renilla luciferase, Promega, Madison, WI, USA). pGL3/SV40-promoter vector (Promega, Madison, WI, USA) and pGL3-Basic vector served as the positive and negative controls, respectively. Each transfection experiment was carried out in triplicate.

After transfection with plasmids for 24 h, the Firefly and Renilla luciferase activities of cells were measured using a Dual-Luciferase Reporter Assay System Kit (Promega, Madison, WI, USA) and VICTOR X2 Multilabel Plate Reader (PerkinElmer, Inc. Waltham, MA, USA). In each well, Firefly luciferase activity was normalized to Renilla luciferase activity. The activity of negative control pGL3-basicwas set to 1.

### 2.10. Statistical Analysis

Real-time PCR results were analyzed by the 2^−ΔΔCt^ method [31]. Statistical evaluation of the data was conducted in SPSS version 15.0 software (SPSS Inc, Chicago, IL, USA). One-way ANOVA test and bivariate correlations were used for statistical analysis. The data are presented as means ± standard deviation (SD) of independent determinations.

## 3. Results

### 3.1. The Tissue Expression of Ovine GDF9

**Figure 1:**
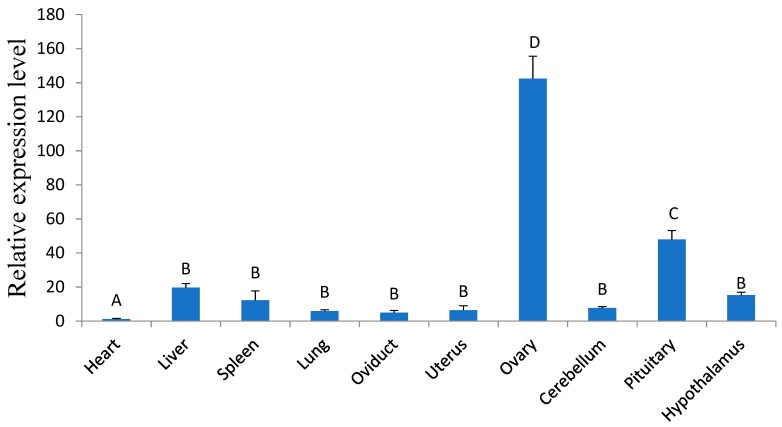
The tissue expression pattern of ovine *GDF9*. The real-time PCR results, the results were expressed as the means ± SD, the different letters above the bars represent significant differences (Relative to the heart group of A, B, and C represent *p* < 0.01, D represents *p* < 0.001. Among B, C, and D differences represent *p* < 0.01).

*GDF9* mRNA levels were quantified using real-time PCR in ten tissues of STH sheep. After real-time PCR, the melting curve had only one peak and the amplification efficiency reached 2.013. The *GDF9* mRNA level in the heart was arbitrarily set to 1. *GDF9* mRNA levels in the ovary were significantly higher (*p* < 0.01) than in other tissues, with lower levels being found in the pituitary, liver, hypothalamus, spleen, cerebellum, uterus, lung, oviduct, and heart (Figure 1).

### 3.2. Analysis of the GDF9 Promoter Region

**Figure 2:**
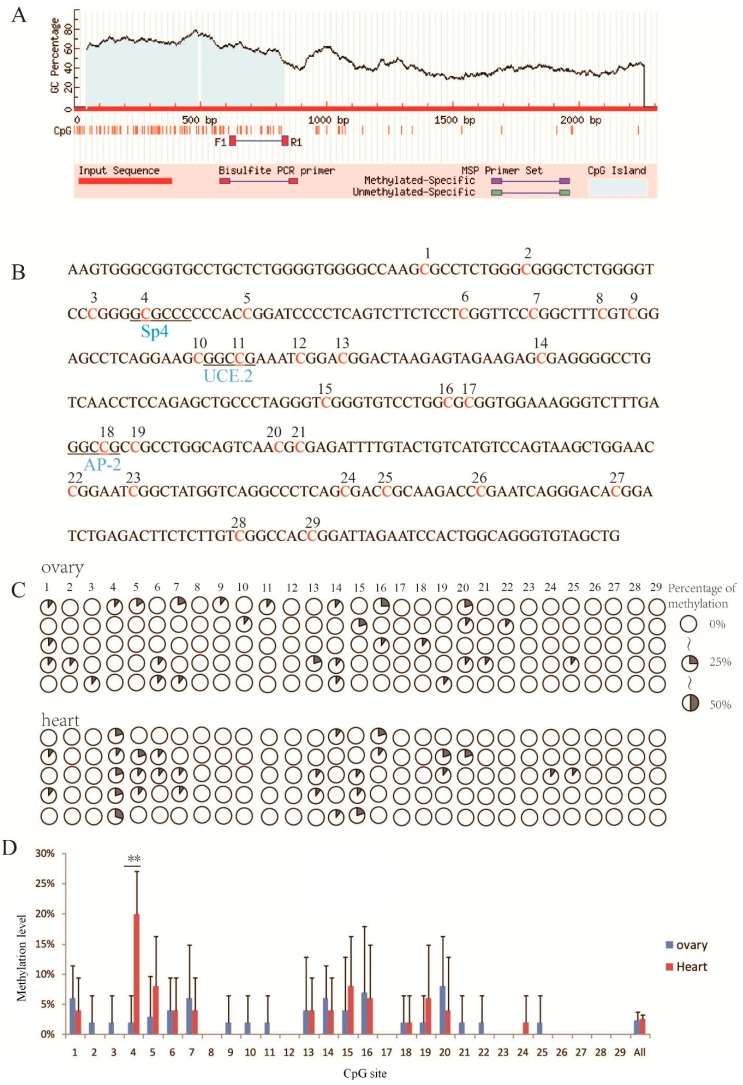
Methylation level of one CpG island (−1453 to −1854) of ovine *GDF9* in ovary and heart tissues. (**A**) The CpG island (sequences from F1 to R1) selected for methylation analysis. (**B**) The CpG site and transcription factor binding sites in this CpG island. A total of 29 CpG sites (each site is numbered above) and three transcription factor binding sites (Sp4, UCE.2, AP-2, each sequence is underlined) are included. (**C**) Methylation levels of CpG sites in ovary and heart. Rows represent tissue from the five STH sheep; columns indicate CpG site. The overall methylation level was relatively low, and the level of mC-4 in heart was relatively high (20%). (**D**) Histogram of methylation level of CpG sites. The mC-4 methylation level was significantly different (** *p* < 0.01) between heart and ovary, and mC-4 was coincidentally present on Sp4 transcription factor binding sites.

Analysis of the promoter revealed two CpG islands. A BSP primer was designed for the CpG island at position −1423 to −1854, as it was closer to the transcription start site (TSS) (Figure 2A). This fragment included 29 CpG sites and three transcription factor binding sites (Sp4, UCE.2, AP-2) (Figure 2B) (Table 2).

### 3.3. Methylation Level

**Figure 3:**
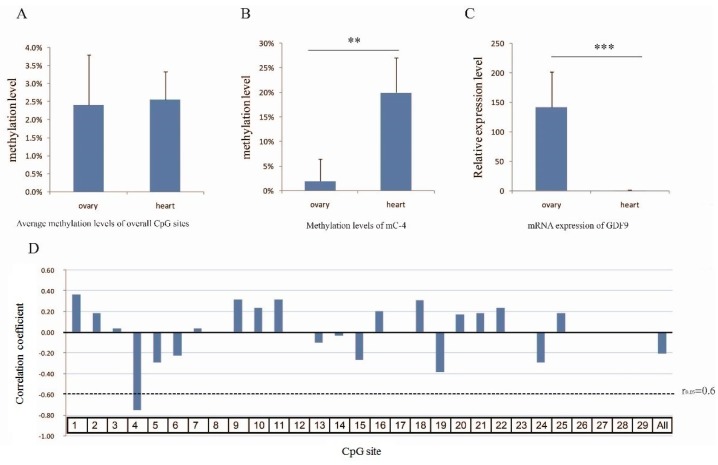
*GDF9* mRNA expression and the correlation with methylation level. (**A**) The average methylation level of overall CpG sites, with no significant difference between heart and ovary. (**B**) The methylation level of mC-4 in ovary and heart, significantly lower in ovary than that in heart (** *p* < 0.01). (**C**) The mRNA expression of *GDF9* in ovary and heart, with significantly higher expression in ovary than that in heart (*** *p* < 0.001). (**D**) Pearson’s correlation coefficient for correlations between mRNA expression and methylation level. mRNA level was significantly correlated with methylation at the mC-4 site. Dashed line (r_0.05_) represents the correlation coefficient threshold.

All CpG sites had low methylation levels (0–30%), and the average level was only 2.52% (Figure 2C). However, the mC-4 level in heart (20%) was significantly higher than that of other sites in either tissue type (Figure 2D) (*p* < 0.01). Interestingly, mC-4 corresponded to a Sp4 transcription factor binding site (Figure 2B). The overall methylation level between heart and ovary was not significantly different (*p* > 0.05) (Figure 3A), but the mC-4 level in the ovary was significantly lower (*p* < 0.01) than that in heart (Figure 3B).

### 3.4. Correlation between the Methylation Level and mRNA Expression

The expression of *GDF9* was significantly higher in ovary than that in heart (Figure 3C) (*p* < 0.001). As Figure 3D showed, although the methylation level of CpG island was negatively correlated with *GDF9* mRNA level (R = −0.21, r_0.05_ = 0.60), only the mC-4 site showed a significant correlation coefficient (R = −0.75, *p* = 0.012) (Figure 3D).

### 3.5. Identification of the Core Region of the Ovine GDF9

**Figure 4:**
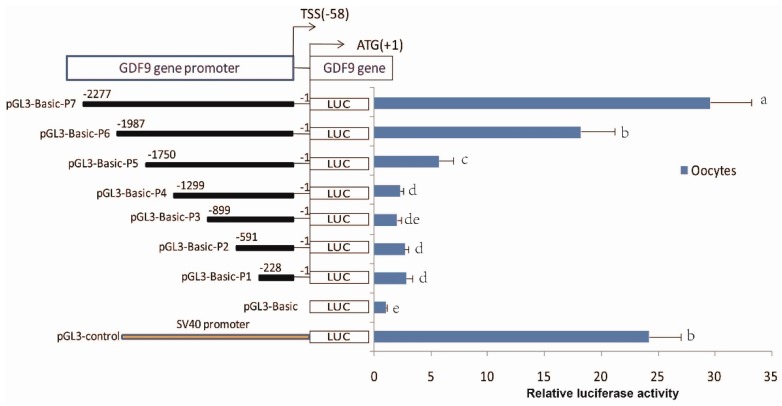
Identification of the core region of the ovine *GDF9* promoter. Truncated 5′-flanking sequences of the *GDF9* are labeled on the left panel and include pGL3-basic-P1 (−1/−228), pGL3-basic-P2 (−1/−591), pGL3-basic-P3 (−1/−899), pGL3-basic-P4 (−1/−1299), pGL3-basic-P5 (−1/−1750), pGL3-basic-P6 (−1/−1987), and pGL3-basic-P7 (−1/−2277). The luciferase activities of the promoter fragments in oocytes are shown on the right panel. Data of three independent replicates are presented as means ± SD. Each of the adjacent lowercase letters represent significant differences (*p* < 0.05).

All deletion constructs showed an increase in luciferase activity when compared with that of the pGL3-basic negative control. A significant increase (*p* < 0.05) of luciferase activity was observed in pGL3-basic-P6 (−1/−1987), when compared to pGL3-basic-P5 (−1/−1750), revealed that the core region of the *GDF9* promoter was located between −1987 and −1750 (Figure 4). Interestingly, the mC-4 site (−1790) occurred in this region. In addition, pGL3-basic-P7 showed significantly higher luciferase activity than that of pGL3-basic-P6 (*p* < 0.05), suggesting the region of −1987 to −2277 promoted relative luciferase activity.

### 3.6. Verification of the Effect of the mC-4 Site

**Figure 5:**
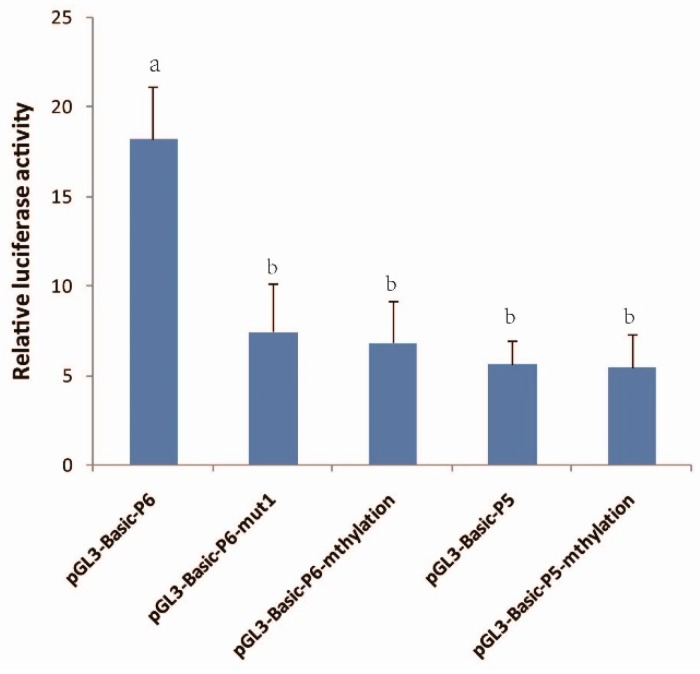
Verification of the effect of the mC-4 site by dual-luciferase. pGL3-Basic-P6-mut1 is site-directed mutation of mC-4; pGL3-Basic-P6-mthylation and pGL3-Basic-P5-methylation are under vitro-methylation; pGL3-basic-P5 and pGL3-basic-P6 are controls. Bars indicate means ± SD. Different superscripts indicate significant difference (*p* < 0.05).

Constructs mutated at the mC-4 site (pGL3-Basic-P6-mut1) and in vitro-methylation (pGL3-Basic-P6-mthylation) both showed similar luciferase activity and resulted in a 50% reduction (*p* < 0.05) in promoter activity in comparison to that of wild type sequence (pGL3-Basic-P6), indicating that the mC-4 site affected the activity of *GDF9* promoter (Figure 5). To control for the effects of methylation in other regions, a luciferase assay was performed in pGL3-Basic-P5 and pGL3-Basic-P5-methylationtransfected cells, and found that those constructs had similar luciferase activity, implying that methylation has no effect on the region of −1 to −1750 (Figure 5).

## 4. Discussion

The tissue expression pattern of *GDF9* has been examined in many animals, with high expression levels in ovary [14,32,33]. In this study, ovine *GDF9* expression was explored by real-time PCR, and *GDF9* expression was significantly higher in ovary than in other tissues. This finding was similar to results from former studies using RT-PCR, RNA-sequencing, and FISH [15,16,17,22] and reinforces that *GDF9* expressionis tissue-specific in sheep.

The underlying functional mechanisms contributing to the tissue-specific expression pattern of *GDF9* remain unclear. A study found that a distal promoter NOBOX binding element (−1881) can enhance expression of *GDF9* in buffalo oocytes, therefore resulting in cell-specific expression [30]. However, ovine *GDF9* lacks a NOBOX binding element, which implies that sheep may have a different regulation mechanism when compared to cattle.

Methylation is one of the most common mechanisms by which tissue-specific expression is regulated. Many genes exhibit tissue-specific expression due to the methylation of the 5′-flanking sequence, especially the CpG island [27,34,35]. In this study, methylation levels of CpG island (−1453 to −1854) were relatively low with no difference between heart and ovary, but mC-4 exhibited significantly higher methylation levels in heart than in ovary. Whole genome bisulfite sequencing revealed that most of the genes, with the exception of silent genes, had low methylation levels in the 5′-flanking sequence [36]. Accordingly, *GDF9* still had low expression levels in the heart, indicating that it is not a silent gene. Furthermore, DNA methylation of some important transcription factor binding sites, even given low methylation levels, can influence the expression of gene [27].

Interestingly, mC-4 presented on Sp4 transcription factor binding sites and the methylation level of this site were negatively correlated with the mRNA expression. Additionally, mutation or methylation of mC-4 can significantly reduce basal transcriptional activity of the *GDF9* promoter in oocytes (*p* < 0.05). Sp4, a member of the Sp1-family of zinc finger transcription factors, is required for normal murine male fertility [37]. Male Sp4^null^ mice do not breed, and female Sp4^null^ mice have a smaller uterus and they exhibit a pronounced delay in sexual maturation [38], which implies that Sp4 plays an important role in reproduction. Therefore, it is possible that Sp4 regulates *GDF9* expression, thereby influencing reproduction. In this study, the ovary was characterized by high *GDF9* expression with low methylation levels of mC-4, while the opposite was observed in the heart. The methylation at the mC-4 site may inhibit the DNA-binding capacity of Sp4 and reduce *GDF9* expression.

Finally, it is possible that additional sites may also influence gene expression because only one fragment of CpG island was analyzed, and other fragments (Figure 2A) still require further investigation. Furthermore, the region of −1987/−2277 promoted the relative luciferase activity. Notably, three estrogen response elements (ERRG, ER2, ERRB) (Table 2) were detected in this region, and this binding site could be important for folliculogenesis.

## 5. Conclusions

In summary, the methylation status of the ovine *GDF9* promoter CpG island mC-4 site might affect the binding of the Sp4 transcription factor, thereby regulating *GDF9* expression and resulting in tissue-specific expression. This study provides useful information for understanding the role of GDF9 gene in the reproduction of sheep. 

## Figures and Tables

**Table 1:** Primers used in this study.

Primer	Primer Sequence (5′→3′)	Annealing Temperature (°C)	Amplified DNA Fragment (bp)
Primers used in real-time PCR			
GDF9-YG-F	CAGACGCCACCTCTACAACA	60	197
GDF9-YG-R	CAGGAAAGGGAAAAGAAATGG		
GAPDH-F	GAGAAACCTGCCAAGTATGA	60	139
GAPDH-R	CGAAGGTAGAAGAGTGAGTG		
Primers used in promoter activity analysis			
P1F	CGGGGTACCACTATATGGCCAAGTAAATCTGAATC	58	228
P2F	CGGGGTACCGTTCTCTGCTCTCTGGAATCTCAATTTC	58	591
P3F	CGGGGTACCCCTGATCTTAGCTCAGAGGCAAGAAC	58	899
P4F	CGGGGTACCTGTCATGTTGCCCACTGTTCACTGCC	58	1299
P5F	CGGGGTACCGGCTTTCGTCGGAG	58	1750
P6F	CGGGGTACCCCACTTCCGGTAGATCGGACG	58	1987
P7F	CGGGGTACCCTGCTCGAAGGGCGACAAGCTAT	58	2277
PR	GTCGTCAAGCTTGGCTTGGAAGAATTAGCAAGG	-	-
Primers used in methylation analysis			
M1F	GGGATTTGTCGTCGTTAAT	55	352
M1R	CAAAACCCGCCCAAAAAC		

**Table 2:** Identification of the putative cis-regulatory elements upstream of ovine growth differentiation factor 9 (*GDF9*).

TFs	Description	Position	Sequence	Strand
AP-2	Transcription factor AP-2, beta	−1669, −1655	gctGCCCtagggtcg	+
SP4	Sp4 transcription factor	−1796, −1780	tcccgggGCGCCcccca	+
ATF	Activating transcription factor 1	−1867, −1847	cccacttcACGTcacgcggcg	−
EBOX	E-box binding factors	−1869, −1853	cccgccgCGTGacgtga	+
USF	Upstream stimulating factor 1	−1870, −1854	cacgTCACgcggcgggg	−
CREB	cAMP-responsive element binding protein	−1916, −1896	gtctccaggTGACggcgccat	+
ERRG	Estrogen-related receptor gamma binding site	−1960, −1938	ccaggaggcggtgaGGTCacttc	+
ER2	Estrogen receptor 2 (ER beta)	−2034, −2016	cgagGTCActtcgcccact	+
ERRB	Estrogen-related receptor beta	−2045, −2023	cactcacacaccgAGGTcacttc	+
